# dbNSFP: A Lightweight Database of Human Nonsynonymous SNPs and Their Functional Predictions

**DOI:** 10.1002/humu.21517

**Published:** 2011-04-21

**Authors:** Xiaoming Liu, Xueqiu Jian, Eric Boerwinkle

**Affiliations:** Human Genetics Center, School of Public Health, The University of Texas Health Science Center at HoustonHouston, Texas

**Keywords:** dbNSFP, functional prediction, database, SIFT, Polyphen2, LRT, MutationTaster, PhyloP

## Abstract

With the advance of sequencing technologies, whole exome sequencing has increasingly been used to identify mutations that cause human diseases, especially rare Mendelian diseases. Among the analysis steps, functional prediction (of being deleterious) plays an important role in filtering or prioritizing nonsynonymous SNP (NS) for further analysis. Unfortunately, different prediction algorithms use different information and each has its own strength and weakness. It has been suggested that investigators should use predictions from multiple algorithms instead of relying on a single one. However, querying predictions from different databases/Web-servers for different algorithms is both tedious and time consuming, especially when dealing with a huge number of NSs identified by exome sequencing. To facilitate the process, we developed dbNSFP (database for nonsynonymous SNPs' functional predictions). It compiles prediction scores from four new and popular algorithms (SIFT, Polyphen2, LRT, and MutationTaster), along with a conservation score (PhyloP) and other related information, for every potential NS in the human genome (a total of 75,931,005). It is the first integrated database of functional predictions from multiple algorithms for the comprehensive collection of human NSs. dbNSFP is freely available for download at http://sites.google.com/site/jpopgen/dbNSFP. Hum Mutat 32:894–899, 2011. © 2011 Wiley-Liss, Inc.

## Introduction

A nonsynonymous SNP (NS) is a single nucleotide variant that causes an amino acid change of its corresponding protein. Because of their direct link to protein changes, NSs are believed to be the major contributor to heritable human diseases among all types of variants in the human genome. As evidence, NSs constitute more than half of the entries of disease-causing genetic changes in the Human Gene Mutation Database (HGMD) [Stenson et al., 2009]. Based on current technologies, it is too costly and time-consuming to investigate the functional effect of every NS experimentally. Fortunately, there are algorithms available to help to predict the potential of a NS being functional or deleterious.

Functional prediction algorithms for NS have been developed in recent years with the advance of phylogenetics, structure biology, bioinformatics and population genetics (see reviews by [Horner et al., 2010; Karchin, 2009; Ng and Henikoff, 2006; Thusberg and Vihinen, 2009]). Usually, the algorithms output a score measuring how likely an NS is deleterious, along with its binary prediction. Functional predictions play an important role in sequencing based genotype–phenotype association analyses. Prediction scores have been used to weight different NSs in order to increase the power of detecting genes influencing a trait [Price et al., 2010]. More importantly, functional prediction has become an indispensable step in identifying genes causing rare Mendelian diseases using an exome-sequencing approach [Cooper et al., 2010; Ng et al., 2010a, b, c]. Functional predictions or conservation information are used to filter or prioritize the novel NSs for further analysis or confirmation [Ng et al., 2010]. Because different algorithms use different information and are based on different training data, each has its own strength and weakness. It has been suggested to use the outputs from multiple algorithms to make a more reliable prediction for a NS (e.g., by using consensus prediction or majority vote) [Chun and Fay, 2009].

Because querying predictions from different databases/Web-servers for different algorithms is both tedious and time consuming, we developed dbNSFP (database for nonsynonymous SNPs' functional predictions) to facilitate the process. We first compiled a collection of all possible NSs in the human genome (a total of 75,931,005) based on the annotation of the Consensus Coding Sequence (CCDS) project [Pruitt et al., 2009]. We next collected their corresponding prediction scores from four new and popular prediction algorithms (SIFT [Kumar et al., 2009], Polyphen2 [Adzhubei et al., 2010], LRT [Chun and Fay, 2009], and MutationTaster [Schwarz et al., 2010]). We also added other related information including a conservation score (PhyloP) [Siepel et al., 2006], degenerate type, and corresponding codons and genes. The dbNSFP is the first known integrated database of functional predictions from multiple algorithms for the comprehensive collection of human NSs.

## Data Sources and Processing

The genes and their corresponding codons were determined based on CCDS version 20090327. This is the latest version based on the human reference sequence build hg18. Although the current human reference sequence is build hg19, many important human sequence resources are based on hg18, including current exome capture chips and the 1,000 genomes pilot data. To facilitate NS queries based on hg19, we used the liftOver tool from the UCSC Genome Browser [Rhead et al., 2010] to convert the coordinates of NSs to hg19. Only 561 NSs out of 75,931,005 (0.0007%) were not converted successfully.

The PhyloP scores were extracted from the placental subset of the precomputed phyloP44way scores [Pollard et al., 2010] provided by the UCSC Genome Browser (see details at http://hgdownload.cse.ucsc.edu/goldenPath/hg18/phyloP44way/). The original score is presented as *phyloP*_*ori*_ = − log_10_*P* or log_10_*P*, if the site is more conserved than neutral (*phyloP*_*ori*_>0) or less conserved than neutral (*phyloP*_*ori*_<0), respectively. *P* is a two-sided *P*-value based on a likelihood ratio test. To make it easier to compare with the prediction scores, we rescaled it to a new score as *phyloP*_*new*_ = 1 − 0.5 × 10^−^*
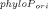
* if *phyloP*_*ori*_>0 or *phyloP*_*new*_ = 0.5 × 10*
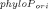
* if *phyloP*_*ori*_<0 to mimic a one-sided *P*-value. The new score ranges from 0 to 1 and a larger score signifies higher conservation. We used *phyloP*_*new*_>0.95 as a rule to predict conserved site [Siepel et al., 2006]. That is, the prediction is “C(onserved)” if *phyloP*_*new*_>0.95; otherwise, the prediction is “N(on-conserved).”

Original SIFT scores were provided by ANNOVAR [Wang et al., 2010], which were originally from a local database format of SIFT 4.0.3. The original SIFT scores (*SIFT*_*ori*_) range from 0 to 1. If a score is smaller than 0.05 the corresponding NS is predicted as “D(amaging)”; otherwise it is predicted as “T(olerated).” We defined a new score *SIFT*_*new*_ = 1 − *SIFT*_*ori*_. The new score still ranges from 0 to 1 and a larger score means more likely to be deleterious. Correspondingly, if a new score is larger than 0.95, the prediction is “D”; otherwise, it is “T.”

Original LRT scores (*LRT*_*ori*_) were downloaded from the LRT Webserver (http://www.genetics.wustl.edu/jflab/lrt_query.html). *LRT*_*ori*_ is a two-sided *P*-value of the likelihood ratio test of codon constraint. Each *LRT*_*ori*_ is associated with an estimated nonsynonymous-to-synonymous-rate ratio (ω) and an amino acid alignment of 31 species at the test codon. If the codon is more constrained than neutral, ω<1; otherwise, ω≥1. We defined our LRT score as *LRT*_*new =*_1 − *LRT*_*ori*_ × 0.5 if ω<1, or *LRT*_*new*_ = *LRT*_*ori*_ × 0.5 if ω≥1 to mimic a one-sided p-value. The score ranges from 0 to 1 and a larger score signifies that the codon is more constrained or a NS is more likely to be deleterious. LRT predictions were derived using the rules summarized in Supp. Figure S1, which is similar to those of Chun and Fay [2009]. In short, (1) a predicted D(eleterious) NS needs to fulfill three requirements: (i) from a codon defined by LRT as significantly constrained (*LRT*_*ori*_<0.001 and ω<1), (ii) from a site with ≥10 eutherian mammals alignments, and (iii) the alternative AA is not presented in any of the eutherian mammals; (2) a predicted N(eutral) NS needs to fulfill either of the two requirements: (i) the alternative AA is presented in at least one of the eutherian mammals, or (ii) from a codon defined by LRT as not significantly constrained (*LRT*_*ori*_≥0.001 or ω≥1) and with ≥10 eutherian mammals alignments; (3) otherwise, the NS is reported as U(nknown). More details of the LRT predictions can be found in the Discussion section.

Polyphen2 scores were manually queried and downloaded as ∼500 batches from its batch query server (http://genetics.bwh.harvard.edu/pph2/bgi.shtml). The default query settings (Classifier model: HumDiv, Transcipts: Canonical, Annotations: Missense) were used except with genome assembly NCBI36/hg18 instead of GRCh37/hg19. We used pph2_prob as our Polyphen2 score, which is the classifier probability of the alternative allele being deleterious. The score ranges from 0 to 1, and the corresponding prediction is “probably damaging” (coded as “D”) if it is larger than 0.85; “possibly damaging” (coded as “P”) if it is between 0.85 and 0.15 and “benign” (coded as “B”) if it is smaller than 0.15. Throughout this article, we regard “D” as its category for deleterious NSs.

MutationTaster scores were queried from its Webserver (http://www.mutationtaster.org/) using its batch query Perl scripts for each of the 75,931,005 NSs. The input file needs an ENSEMBL transcript ID and a sequence snippet (the immediate upstream and downstream sequence from the query site) for each SNP. We first used ANNOVAR to annotate the NSs using ENSEMBL database to get the corresponding ENSEMBL transcript ID for each NS. If there is more than one transcript ID, initially the first ID was used to build an input file for the first round query. If it turned out that transcript ID is not in the MutationTaster's database (as reported in its output file), we then used the second ID to build an input file for the second round of query. Snippets were built using the FASTA files of the human reference sequence downloaded from the UCSC Genome Browser. For the first round query, we used 12 upstream and 12 downstream nucleotides from the query site to build the snippet. If the output file reported that the snippet is not unique, then 100 upstream and 100 downstream nucleotides from the query site were used to build a new snippet for a second round query. MutationTater has four types of predictions: “disease_causing_automatic,” “disease_causing,” “polymorphism,” and “polymorphism_automatic,” which we coded as “A,” “D,” “N,” and “P,” respectively. Among them, “D” and “N” are determined by the prediction algorithm, whereas “A” and “P” are determined by external information (see the Discussion section for details). We regard “A” and “D” as categories for deleterious NSs. MutationTaster also reports a *P*_value for each prediction, which represents the probability that the prediction is true. We defined our MutationTaster score using the following rule: if the prediction is “disease_causing” or “disease_causing_automatic,” score = *P*_value; if the prediction is “polymorphism” or “polymorphism_automatic”, score = 1 − *P*_value. The resulting score ranges from 0 to 1 and a larger score means more likely to be deleterious.

For various reasons, an algorithm may not output a prediction or score for a NS, thus regarded as missing data and reported it as “NA.” In dbNSFP, we reported imputed scores for missing scores (see Missing Data and Imputation). Therefore, a prediction of “NA” can be used as an indicator to determine whether the corresponding score is imputed or not. Rarely, probably due to different gene annotations for the same NS, an algorithm may report a different reference AA or alternative AA as to those defined by CCDS. In that case, we considered the predictions and scores (if reported) as missing data. On the other hand, for a small number of NSs, CCDS may define more than one reference AA or alternative AA for the same nucleotide site change, due to different annotations for the same gene. We treated those definitions as different NSs. In another words, we defined a NS as a unique combination of chromosome, position, reference nucleotide allele, alternative nucleotide allele, reference AA allele, and alternative AA allele.

## Database Contents

In the current version, each NS links to the following entries: chromosome number, physical position on the chromosome as to hg18 (1-based coordinate), reference nucleotide allele (as on the + strand), alternative nucleotide allele (as on the + strand), reference AA, alternative AA, physical position on the chromosome as to hg19 (1-based coordinate), gene name, gene Entrez ID, CCDS ID, reference codon, position on the codon (1, 2, or 3), degenerate type (0, 2, or 3), AA position as to the protein, coding sequence (CDS) strand (+ or − ), estimated nonsynonymous-to-synonymous-rate ratio (ω, reported by LRT), PhyloP score, PhyloP prediction, SIFT score, SIFT prediction, Polyphen2 score, Polyphen2 prediction, LRT score, LRT prediction, MutationTaster score, MutationTaster prediction. If any of the PhyloP, SIFT, Polyphen2, LRT, and MutationTaster scores were missing, their imputed scores (see Missing Data and Imputation) were reported (but their corresponding predictions will be “NA”). There are a total of 75,931,005 entries for 64,646,969 unique NSs, each of which is defined as a unique combination of physical position and alternative allele. This is due to the fact that the same NS may be annotated by CCDS multiple times for different forms of the same gene. The numbers of nonmissing entries of NS, hg19 position, PhyloP score, SIFT score, Polyphen2 score, LRT score, and MutationTaster score per chromosome are summarized in Table 1.

**Table 1:** Number of Entries in dbNSFP

Chromosome	NS	hg19	PhyloP	SIFT	Polyphen2	LRT	MutationTaster
1	8329939	8329939	8329796	7611044	7279405	7644552	7959066
2	5115071	5115063	5114264	4376414	4428042	4765076	4858487
3	4280786	4280782	4280786	3804983	3756644	4063236	4098445
4	2868872	2868872	2868872	2532905	2554593	2752943	2766053
5	3484907	3484907	3484907	3134219	3043592	3207583	3347045
6	4075171	4075036	4075171	3710396	3586226	3824055	3803359
7	3268295	3268295	3267997	2819288	2856409	2989039	3132425
8	2597009	2597009	2597009	2278629	2207857	2456441	2539459
9	3164462	3164462	3164462	2893822	2842191	2902636	2860881
10	3273027	3273027	3273027	2924577	2922389	3093339	2956250
11	4253046	4253046	4253046	3793927	3795848	4004874	4098876
12	3852960	3852960	3852960	3412527	3332085	3698888	3723502
13	1420096	1420096	1420096	1278533	1235036	1378650	1374240
14	2494133	2494133	2494133	2262099	2236525	2383704	2432185
15	2470042	2469767	2470042	2206987	2219513	2317477	2351630
16	2970643	2970643	2970623	2589515	2545952	2794456	2864645
17	4372234	4372095	4371639	3943103	3822645	4019068	4228681
18	1178377	1178377	1178377	1031129	1039874	1090517	1131942
19	4415600	4415600	4415600	3916565	3779650	3360865	3983007
20	2103098	2103098	2103098	1935033	1881731	1998568	1927606
21	989231	989231	989231	843131	842902	890113	925335
22	1553703	1553703	1553703	1388173	1338949	1444075	1461233
X	3176068	3176068	3175174	2941495	2719583	2834074	2998558
Y	224235	224235	223727	205265	195144	138312	146802
Total	75931005	75930444	75927740	67833759	66462785	70052541	71969712
Total (unique)^a^	64646969	64646408	64643749	57471955	56620320	59419546	61277012

a NSs with the same position and alternative allele were counted only once.

**Figure 1:**
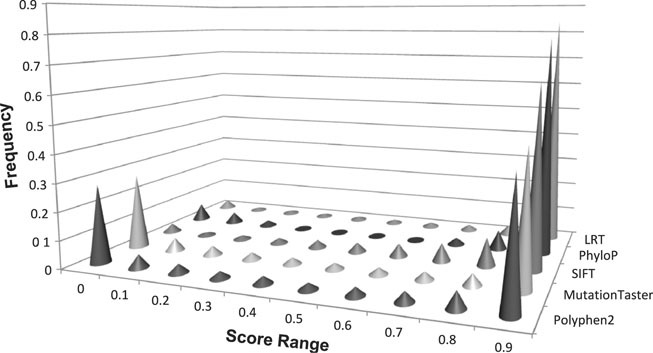
Distributions of PhyloP, SIFT, Polyphen2, LRT, and MutationTaster scores.

Because all scores (PhyloP, SIFT, Polyphen2, LRT, and MutationTaster) were scaled to [0, 1] with a larger score corresponding to higher conservation (PhyloP and LRT) or more likely to be deleterious (SIFT, Polyphen2, and MutationTaster), we can easily compare their distributions. Figure 1 shows the histograms of their total scores in different score ranges. An interesting observation is that all five methods show some form of bimodal distribution, with the majority of the scores clustered around 1 and a small proportion of the scores clustered around 0. The predictions from Polyphen2 and MutationTaster are more balanced, which have about 30% of the scores in range [0, 0.1), whereas the remaining three only have less than 7% scores in that range. Correspondingly, SIFT, LRT, and MutationTaster all predict more than half of the total NSs being deleterious, whereas Polyphen2 predicts that slightly less than half of the NSs are deleterious (Table 2). The proportion of pairwise agreement between methods on whether aN NS is deleterious is around 60–70%, with the highest agreement between the LRT and MutationTaster (77.19%) (Table 3). The Spearman's rank correlation coefficients (RCCs) between scores of different methods show low to moderate positive correlations, with the highest RCC between LRT and MutationTaster being 0.62 (Table 3). We also calculated the Pearson's correlation coefficients (data not shown) for each pair of the methods. They are all smaller than their corresponding RCCs, which suggests stronger nonlinear correlation between those scores.

**Table 2:** Summary of Predictions

Method	SIFT	Polyphen2	LRT	MutationTaster
Unknown/missing	8097246	9468220	10556339	3961293
Nondeleterious	31166227	35477555	25950325	29158468
Deleterious	36667532	30985230	39424341	42811244

**Table 3:** Pairwise Prediction Agreement Percentages (Upper Right Triangle) and Spearman's Rank Correlation Coefficients (Lower Left Triangle)

Method	PhyloP	SIFT	Polyphen2	LRT	MutationTaster
PhyloP	–	–	–	–	–
SIFT	0.189	–	68.82	62.14	61.69
Polyphen2	0.306	0.517	–	66.82	64.91
LRT	0.475	0.267	0.457	–	77.19
MutationTaster	0.389	0.313	0.444	0.618	–

## Missing Data and Imputation

The numbers of missing data of PhyloP, SIFT, Polyphen2, LRT, and MutationTaster per chromosome can be derived from Table 1. Among them, PhyloP has the lowest missing data rate (0.004%). As for the four prediction methods, MutationTaster has the lowest missing data rate (5.2%), followed by LRT (7.7%), SIFT (10.7%), and Polyphen2 (12.5%). This is partially because SIFT and Polyphen2 do not provide prediction scores for stop-gain (a mutation that changes an AA codon to a stop codon) or stop-loss (a mutation that changes a stop codon to an AA codon) and LRT does not provide prediction scores for stop-loss.

An advantage of having multiple prediction scores for the same NS is that it facilitates better imputation for missing scores. Some statistical methods for detecting association between (groups) of rare variants and phenotypes (e.g., VT test) [Price et al., 2010] use prediction scores to weight NSs to increase their detecting power. If scores are missing, typically they are imputed using the average of the nonmissing scores (of the same algorithm) in the sample. As we observed in Figure 1, the prediction scores are bimodal distributed. Therefore, using the average score to impute may not be a good choice. Although there are only low to moderate correlations between different types of scores, it is still possible to make full use of those relationships to obtain better imputation scores.

**Figure 2:**
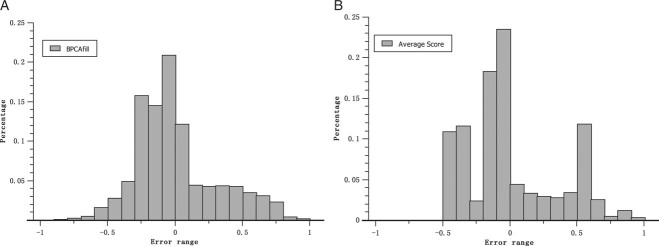
Distributions of imputation errors by using BPCAfill (**A**) or using the average score of the same algorithm (**B**).

We chose the program BPCAfill [Oba et al., 2003] to impute the missing scores in dbNSFP, because of its higher accuracy and faster computation comparing to other methods [Aittokallio, 2010]. Although BPCAfill is originally designed for imputing missing expression data for microarray analyses, it can be applied to any data set with correlated columns or rows. To evaluate the performance, (1) we compiled a subset of dbNSFP, in which all five scores are NOT missing; (2) for each missing pattern observed in dbNSFP, we randomly picked the same number of NSs as those with the missing pattern in dbNSFP, and marked their corresponding scores as missing (e.g., dbNSFP has 334,421 NSs with LRT and MutationTaster scores missing while all other three scores are present; we randomly picked 334,421 NSs from the subset and marked their LRT and MutationTaster scores as missing); and (3) after BPCAfill imputed the missing scores, we compared the imputed scores with the corresponding original scores. Figure 2 shows the histograms of the error (imputed score minus original score) distribution from two different imputation strategies: imputing with BPCAfill or with the average scores of the same algorithm. Imputation errors for each algorithm with different missing patterns are summarized in Supp. Table S1. Comparing the imputation accuracies for different prediction scores over all missing patterns, LRT has the smallest mean squared error (MSE) of 0.035, followed by SIFT (0.054) and PhyloP (0.066), whereas MutationTaster and Polyphen2 have the largest MSEs of 0.133 and 0.142, respectively (Supp. Table S1). A *t*-test of the difference between mean error and 0 shows that imputations with both BPCAfill and the average scores were unbiased (data not shown), with average errors equal to − 6.25 × 10^−6^ (BPCAfill) and −3.6 × 10^−5^ (average score), respectively. However, the distribution of the errors using BPCAfill had a significantly smaller standard deviation than the other strategy (0.303 comparing to 0.341, *P*-value<10^−14^, *F-*test, one tailed). Therefore, we concluded that BPCAfill can produce significantly better imputation scores than simply imputing with the average score.

## Companion Search Program

A companion search program written in Java (search_dbNSFP) is freely available for download along with dbNSFP. It can run crossplatform on a wide range of computers, as long as a proper Java Runtime Environment is installed. It enables a simple search for a NS, a chromosome position or a gene. It runs on a command line with a basic format “java search_dbNSFP -i infile -o outfile,” where infile and outfile are the user designated input file name and output file name, respectively. The input file contains one or more lines, with each line representing a query. The query formats for NS, chromosome position and gene are presented in Table 4. The output file contains all NSs that match the query information. For example, the output of a gene query will contain all NSs in that gene, and that of a genome position query will contain all NSs on that position. If there is more than one result for a NS query due to different annotations (see Data Sources and Processing), all results will be reported. By default, the program searches all chromosomes with the positions according to the human genome reference sequence hg18. Users can specify both the chromosomes to search for and the reference sequence version by using the format “java search_dbNSFP [-v reference_version] [-c chromosome_list] -i infile -o outfile,” where the contents in [ ] are optional. reference_version can be either hg18 or hg19. chromosome_list is a list of chromosome number (1, 2,…, X, Y) separated by commas without space, for example “1,3,22,X.” If there are some queries that do not have matched NS, those failed queries will be listed in the system output (command line environment).

**Table 4:** Format of Queries for Input File

Query type	Format^a^	Example
NS	chr pos ref alt	Y 140855 A C
	chr pos ref alt refAA altAA	Y 140855 A C M L
Genome position	chr pos	10 94454459
Gene	gene_name	PLCXD1
	gene_id	55344
	CCDS_id	CCDS14103.1

a Separated by tab or space. chr: chromosome number; pos: position on chromosome; ref: reference allele; alt: alternative allele; refAA: reference amino acid; altAA: alternative amino acid; gene_name: gene name; gene_id: gene Entrez ID; CCDS_id: CCDS ID.

## Discussion

dbNSFP is a database for all potential NSs (as to the reference sequence) in the human genome and their functional predictions. It was developed for two main purposes. The first is to facilitate the SNP filtering/prioritizing step in studies of mapping rare Mendelian disease genes using the exome-sequencing approach. The second is to facilitate the imputation of missing scores that are required in the weighted sum test (e.g., VT test) [Price et al., 2010] for detecting association between phenotypes and groups of rare variants. A companion search program is provided for fast local queries. For the current version, we attempted to keep the database simple and lightweight to ensure storage and running efficiency. Except for five prediction/conservation scores (PhyloP, SIFT, Polyphen2, LRT, and MutationTaster) for each NS, the database only contains information of its corresponding genomic position, gene, codon, among others. Future development may include expanding the collection of NSs from other genome databases, incorporating the results from other prediction algorithms that do not provide genome position searches, such as PhD-SNP [Capriotti et al., 2006] and PANTHER [Thomas et al., 2003], and building a fully functional Webserver for on-line database query. We plan to keep updating the database to have newer CCDS versions, whenever most of the public available human genome sequence data shift to a new human genome reference sequence.

Some cautions are needed when compiling and interpreting the search results from dbNSFP. First, the NSs collected in dbNSFP were based on the annotations of CCDS, which defines consensus CDSs from multiple genome databases. Therefore, CCDS by no means includes the complete set of human CDSs, but rather includes a set of well-annotated CDSs. It is quite possible that some NSs defined by other databases are not contained in dbNSFP. Second, the NSs in dbNSFP are defined as the alternative alleles against the reference alleles of the human reference sequence. However, the reference allele is neither necessary the “wild-type” allele, nor the common allele in all human populations. Some other information about the alternative allele, for example, its frequency in populations or sampled individuals, may help to determine its potential to be deleterious. Third, for various reasons, some scores are missing for some NSs and the imputed scores may not be reliable. For example, scores for stop-gain NSs are not available from SIFT and Polyphen2. Although imputed scores for stop-gain NSs are provided, some researchers may prefer to assign a score based on their knowledge of the protein. For instance, a stop-gain near the beginning of the protein is more likely to be deleterious than one near the end of the protein. Fourth, there may be multiple entries in dbNSFP that match the same NS query. This is primarily due to the fact that CCDS contains different annotations for different forms of the same gene.

Our rules for LRT predictions (Supp. Fig. S1) are similar to those of Chun and Fay [2009], but be aware that our predictions may not be the same as those reported by the LRT Webserver (http://www.genetics.wustl.edu/jflab/lrt_query.html). The major difference is that the Webserver predicts a NS as either D(eleterious) or N(eutral) according to *LRT*_*ori*_ and ω, regardless of the quality of the underlying amino acid alignment. Chun and Fay [2009] suggested that if the amino acid alignment has less than 10 eutherian mammals, the prediction power is low. Therefore, we add a new prediction category U(nknown) specifically for the low-power situation. As a result, our LRT predictions for D(eleterious) and N(eutral) are more stringent than those provided by the Webserver.

Because the predictions of LRT and MutationTaster are not entirely dependent on the prediction scores, discrepancy between a score and its corresponding prediction may occur. As to LRT, the discrepancies come from N(eutral) predictions with high scores (i.e., the codon is highly constrained or a NS is likely to be deleterious). This is because of one prediction rule: if the alternative AA allele is observed in at least one eutherian mammal, the NS is predicted as N(eutral), regardless of the score it associated with (Supp. Fig. S1). The discrepancy cases of MutationTaster come from the two “automatic” predictions, that is, “disease_causing_automatic” (“A”) and “polymorphism_automatic” (“P”). An NS will be predicted to be “A” if it causes nonsense-mediated decay (i.e., a stop-gain); and an NS will be predicted to be “P” if all three genotypes of the reference/alternative alleles are observed in the HapMap data [The International HapMap Consortium, 2010]. Both types of prediction are not dependent on the prediction scores at all, although the scores are still reported.

As to the missing data imputation, our results clearly showed that BPCAfill can obtain more accurate imputation compared to simply using the average score of the same algorithm (column-average). This is easy to understand because BPCAfill borrows information from the correlated scores of different algorithm while column-average imputation does not use such information. To further evaluate the performance of BPCAfill, we also compared its results to the simple imputation using the average of the nonmissing scores of the other algorithms for the same NS (row-average). For example, if an NS misses a SIFT score while all other four scores (PhyloP, Polyphen2, LRT, MutationTaster) are available, we impute the SIFT score using the average of the other four scores. The results showed that although the row-average imputation's mean absolute error is smaller than that of the column-average, it is still larger than that of BPCAfill (data not shown). It is also shown that the row-average imputation is significantly biased and has a significantly larger variance than BPCAfill (data not shown). The superior performance of BPCAfill compared to the row-average can be explained by the nonlinear correlation between the scores of different algorithms and the fact that BPCAfill also borrows information from the correlation between NSs while row-average imputation does not.

Collecting predictions and scores from multiple algorithms has many advantages. Besides providing better imputation for missing data, as we mentioned in the introduction, it also helps to get more accurate functional predictions for NS. A simple way to incorporate multiple predictions is to rank each NS according to the number of algorithms that predict it as deleterious. The larger the number, the higher the confidence that it is truly deleterious. A better solution is to develop a unified score (or a score of scores) based on training data sets and infer predictions from it. Besides NS, other types of variants may also contribute to diseases. However, the functional prediction methods of those variants are typically less mature than those of NS, but never the less they still provide valuable information of those variants. For example, a recent study [Waldman et al., 2010] showed that codon bias is significantly correlated with gene expression levels in human. Therefore, some synonymous changes are more likely to have strong impact on gene expression and lead to phenotypic effects. Other information that can be used to prioritize variants including the prediction of promoter regions, linkage disequilibrium with known genome-wide association study (GWAS) hits, gene expression profile of disease related tissues, known biochemical pathways, known or predicted gene interaction networks, among others. In recent years, some Webservers have been developed to integrate searches or results from multiple bioinformatics tools, for example, the SeattleSeq Annotation server, F-SNP [Lee and Shatkay, 2008], SNPLogic [Pico et al., 2009], SNPit [Shen et al., 2009], and pfSNP [Wang et al., 2011]. As the field moves from whole exome sequencing to whole genome sequencing, more efforts are needed in these nonprotein coding domains.
