# Supplementary material for: dbNSFP: A Lightweight Database of Human Nonsynonymous SNPs and Their Functional Predictions
Source: Hum Mutat. 2011 Apr 21;32(8):894–9. doi: 10.1002/humu.21517 (PMC3145015; doi:10.1002/humu.21517)
Supplement: Supplementary file 1 [file humu0032-0894-SD1.pdf]

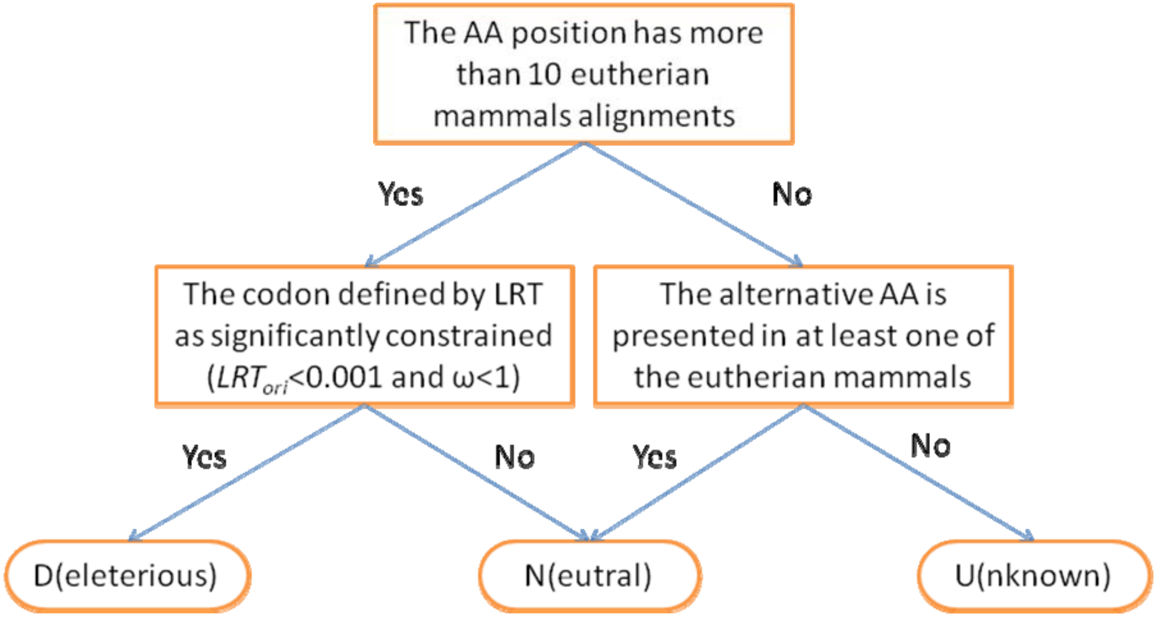

Supp. Figure S1. LRT prediction rules.

Supp. Table S1. Summary of imputation errors for each algorithm with different missing patterns

Missing pattern[1]	PhyloP	SIFT	Polyphen2	LRT	MutationTaster	Average
"00000"	-0.0356 (0.222)[2]	0.0127 (0.303)	0.0776 (0.420)	-0.0156 (0.124)	-0.0465 (0.437)	-0.00147 (0.327)
"00001"	-0.00747 (0.274)	0.0147 (0.263)	-0.0284 (0.419)	0.00799 (0.223)	NA	-0.00328 (0.304)
"00010"	-0.0407 (0.246)	-0.0281 (0.252)	-0.0438 (0.406)	NA	-0.141 (0.415)	-0.0634 (0.342)
"00011"	-0.0223 (0.213)	0.0857 (0.292)	-0.0963 (0.413)	NA	NA	-0.0110 (0.326)
"00110"	0.135 (0.403)	-0.0124 (0.222)	NA	NA	-0.133 (0.264)	-0.00361 (0.325)
"01000"	0.0130 (0.280)	NA	-0.00360 (0.395)	0.0119 (0.229)	0.0394 (0.405)	0.0152 (0.336)
"01001"	0.000298 (0.258)	NA	0.00829 (0.369)	-0.00466 (0.202)	NA	0.00131 (0.285)
"01010"	-0.0240 (0.214)	NA	0.0497 (0.393)	NA	-0.0186 (0.341)	0.00235 (0.327)
"01011"	0.00962 (0.237)	NA	-0.0293 (0.346)	NA	NA	-0.00984 (0.297)
"01100"	-0.00860 (0.232)	NA	NA	0.0316 (0.259)	0.0619 (0.408)	0.0283 (0.311)
"01101"	0.00976 (0.268)	NA	NA	0.00913 (0.216)	NA	0.00944 (0.243)
"01110"	-0.00750 (0.221)	NA	NA	NA	-0.0330 (0.320)	-0.0203 (0.275)
"10000"	NA	-0.000641 (0.242)	-0.00163 (0.411)	-0.000544 (0.196)	-3.29e-06 (0.400)	-0.000703 (0.326)
"10001"	NA	0.000101 (0.237)	0.000269 (0.387)	-0.000179 (0.187)	NA	6.34e-05 (0.283)
"10010"	NA	6.19e-05 (0.242)	0.000342 (0.403)	NA	-0.000422 (0.378)	-6.06e-06 (0.348)
"10011"	NA	0.000203 (0.237)	-2.73e-06 (0.385)	NA	NA	0.000100 (0.320)
"10100"	NA	-0.000895 (0.223)	NA	0.000119 (0.195)	-0.00190 (0.377)	-0.000891 (0.277)
"10101"	NA	0.000353 (0.223)	NA	0.000269 (0.186)	NA	0.000311 (0.206)
"10110"	NA	0.000196 (0.224)	NA	NA	-0.000645 (0.361)	-0.000224 (0.300)
"10111"	NA	-4.76e-05 (0.222)	NA	NA	NA	-4.76e-05 (0.222)
"11000"	NA	NA	-0.000423 (0.379)	0.000994 (0.197)	0.00160 (0.390)	0.000724 (0.334)
"11001"	NA	NA	-0.000260 (0.363)	-0.000362 (0.187)	NA	-0.000311 (0.289)

**Missing pattern**[1]	**PhyloP**	**SIFT**	**Polyphen2**	**LRT**	**MutationTaster**	**Average**
"11010"	NA	NA	0.000651 (0.373)	NA	-0.000476 (0.369)	8.78e-05 (0.371)
"11011"	NA	NA	2.33e-05 (0.362)	NA	NA	2.33e-05 (0.362)
"11100"	NA	NA	NA	-0.000732 (0.193)	-0.00133 (0.375)	-0.00103 (0.298)
"11101"	NA	NA	NA	1.23e-05 (0.186)	NA	1.23e-05 (0.186)
"11110"	NA	NA	NA	NA	5.14e-05 (0.359)	5.14e-05 (0.359)
Overall	0.00465 (0.257)	0.000107 (0.232)	-5.02e-06 (0.377)	-7.68e-05 (0.187)	-0.000140 (0.365)	-6.25e-06 (0.303)

[1]Missing pattern is a serial set of indicator variables of 0 or 1 corresponding to (from the first to the last) PhyloP, SIFT, Polyphen2, LRT and MutationTaster scores, respectively, where 0 signifies missing/imputed and 1 signifies non-missing/original.
[2]The values outside the parentheses are the means and those inside the parentheses are the standard deviations.
